# Radiation pneumonitis after definitive concurrent chemoradiotherapy with cisplatin/docetaxel for non‐small cell lung cancer: Analysis of dose‐volume parameters

**DOI:** 10.1002/cam4.3093

**Published:** 2020-05-04

**Authors:** Kuniaki Katsui, Takeshi Ogata, Kenta Watanabe, Norihisa Katayama, Masahiro Kuroda, Katsuyuki Kiura, Takao Hiraki, Yoshinobu Maeda, Shinichi Toyooka, Susumu Kanazawa

**Affiliations:** ^1^ Department of Proton Beam Therapy Okayama University Graduate School of Medicine, Dentistry and Pharmaceutical Science Okayama Japan; ^2^ Department of Radiology Iwakuni Clinical Center Yamaguchi Japan; ^3^ Department of Radiology Okayama University Hospital Okayama Japan; ^4^ Department of Radiological Technology Graduate School of Health Sciences Okayama University Okayama Japan; ^5^ Department of Allergy and Respiratory Medicine Okayama University Hospital Okayama Japan; ^6^ Department of Radiology Okayama University Graduate School of Medicine, Dentistry and Pharmaceutical Science Okayama Japan; ^7^ Department of Hematology, Oncology, and Respiratory Medicine Okayama University Graduate School of Medicine, Dentistry and Pharmaceutical Science Okayama Japan; ^8^ Departments of General Thoracic Surgery and Breast and Endocrinological Surgery Okayama University Graduate School of Medicine, Dentistry and Pharmaceutical Science Okayama Japan

**Keywords:** cisplatin/docetaxel, dose‐volume histogram, non‐small cell lung cancer, PACIFIC trial, radiation pneumonitis

## Abstract

**Background:**

Radiation pneumonitis (RP) is a major pulmonary adverse event of chest radiotherapy. The PACIFIC trial that identified durvalumab as an effective subsequent‐line therapy after concurrent chemoradiotherapy (CCRT) found that patients with grade 2 or higher RP may have to be excluded from treatment under certain criteria. The purpose of this study was to investigate the relationship between grade ≥2 RP and the parameters of dose‐volume histograms after CCRT with cisplatin/docetaxel for stage III non‐small cell lung cancer and conduct a subset analysis of severe RP that can lead to the permanent discontinuation of treatment per the PACIFIC trial criteria to help determine treatment strategy.

**Methods:**

We calculated the percentage of the lung volume received at least 5 Gy (V5) and 20 Gy (V20), the mean lung dose (MLD), and the lung volume spared from a 5 Gy dose (VS5) to the total lung volume. Factors affecting the incidence of grade ≥2 RP were identified; severe RP was defined as grade ≥3 as well as grade 2 RP that required ≥10 mg prednisolone for at least 12 weeks.

**Results:**

This study included 45 patients. On univariate analysis, all parameters and total lung volume were found to be significant predictors of grade ≥2 RP (*P* = .001, .003, .03, .004, and .02, respectively). On multivariate analysis, V20 was a significant predictive factor of grade ≥2 RP (*P* = .007). Severe RP developed in 6 of 37 patients (16.2%) whose V20 values were 35% or lower. On univariate analysis, only V20 was a significant predictor of severe RP in these patients (*P* = .01).

**Conclusions:**

The best approach to reduce the rate of grade ≥2 RP is to maintain the V5, V20, MLD, and VS5 as low as possible during radiotherapy planning in patients receiving definitive CCRT with cisplatin/docetaxel.

## Graphical abstract

V20 was a significant predictor of grade ≥2 radiation pneumonitis after chemoradiotherapy with cisplatin/docetaxel for stage III non‐small cell lung cancer. Severe radiation pneumonitis that would lead to the permanent discontinuation of treatment per the PACIFIC trial criteria developed in 6 of 37 patients (16.2%) whose V20 values were 35% or lower. Only the V20 was a significant predictor of severe radiation pneumonitis.

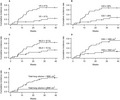

## 1 BACKGROUND

Concurrent chemoradiotherapy (CCRT) is considered one of the standard treatments for medically inoperable stage III non‐small cell lung cancer (NSCLC).^1^, ^2^, ^3^, ^4^ The standard chemotherapy that is administered simultaneously with radiotherapy is a platinum/taxane combination. After CCRT with cisplatin/docetaxel previously showed favorable results,^1^ this regimen has since been the recommended chemotherapy in Japan. Radiation pneumonitis (RP) is a major pulmonary adverse event that can arise from chest radiotherapy; the incidences of grade ≥3 RP ranged between 1.8% and 10.0% in previous prospective studies, while grade 5 RP rates ranged between 0% and 5.6%.^1^, ^2^, ^3^, ^4^ Several studies have clarified the relationship between RP and the parameters of the dose‐volume histogram (DVH) in patients who underwent CCRT.^5^, ^6^, ^7^, ^8^, ^9^, ^10^, ^11^, ^12^, ^13^ These studies found that the lung volume percentage received at least 5 Gy (V5) and 20 Gy (V20), as well as the mean lung dose (MLD), are predictive factors for RP. Moreover Tsujino et al found that the lung volume spared from a 5 Gy dose (VS5)^12^ was also predictive of RP. Another multicenter investigation revealed that the odds ratio of symptomatic RP in patients who received CCRT with carboplatin/paclitaxel was as high as 3.33 when compared with other chemotherapy regimens.^11^ RP significantly occurred more frequently in patients undergoing CCRT with cisplatin/docetaxel than in those receiving concurrent cisplatin/vinorelbine.^8^ Although the concurrent administration of taxanes with radiotherapy is considered a risk factor for RP, no study (to our knowledge) has investigated the correlation between RP and DVH parameters in patients with lung cancer that received CCRT with a single regimen of cisplatin/docetaxel.

Recently, the breakthrough PACIFIC trial studying the administration of durvalumab after CCRT showed that the overall and progression‐free survival rates were significantly higher in patients receiving durvalumab than in those receiving placebo.^14^, ^15^ Since patients who develop grade ≥ 2 RP are no longer eligible for durvalumab, it is crucial to be able to predict the occurrence of grades ≥ 2 RP. Moreover durvalumab should be discontinued if either grade ≥ 3 RP or grade 2 RP that requires >10 mg prednisolone for over 12 weeks develops.^14^ However, the incidences of severe RP that are produced by each type of chemotherapy regimen, as well as factors that predict severe RP, remain unclear.

The aim of this study was to examine the relationship between grades ≥ 2 RP and parameters of DVH in patients with stage III lung cancer who received definitive CCRT with cisplatin/docetaxel and identify predictive factors for the same. Furthermore, we determined the rates of severe RP that would render patients permanently ineligible for continued durvalumab treatment under the criteria of the PACIFIC trial, and also identified DVH‐related risk factors in patients with V20 values <35%.

## 2 METHODS

### 2.1 Patients

We retrospectively evaluated patients who received definitive CCRT between January 2004 and May 2018 at the author's hospital. The inclusion criteria were as follows: stage III NSCLC, a radiotherapy dose of 54 Gy/27 fractions to 66 Gy/33 fractions, cisplatin/docetaxel as concurrent chemotherapy regimen, and no reception of neoadjuvant chemotherapy before the start of radiotherapy. All procedures complied with the ethical standards of the 1964 Declaration of Helsinki and the subsequent amendments. Written informed consent was obtained from all patients prior to treatment. The option to opt‐out was provided to all patients through notifications displayed on the hospital's website and outpatient ward prior to the start of this study. The hospital's review board approved this study.

### 2.2 Treatment

Staging was classified according to the 7th edition of the TNM classification of malignant tumors. Three‐dimensional conformal radiation therapy (3D‐CRT) was performed for all patients. Computed tomography (CT) scans for planning were performed at 2‐10 mm intervals with free‐breathing without four‐dimensional CT scan. The gross tumor volume is a combination of the volume of the primary tumor and clinically diagnosed metastatic lymph node. The margin of clinical target volume was 5‐10 mm. When elective nodal irradiation was performed, non‐metastatic subcarinal and ipsilateral hilar nodal stations were included. The attending physician decided the internal margin based on fluoroscopic images. The margin of planning target volume was 5‐10 mm. The total dose was 54‐66 Gy/27‐33 fractions at isocenter with 10 MV photon beam generated from a linear accelerator (ONCOR, Primus or Mevatron, Canon Medical Systems, Tochigi, Japan). We calculated all plans using the XiO computer software (version 4.8.0, Electa, Stockholm, Sweden) with a non‐uniformity correction algorithm as a superposition. The chemotherapy regimen used concurrently with radiotherapy was cisplatin/docetaxel, based on a prospective trial of OLCSG 0007.^1^

### 2.3 Statistical analysis and evaluation

Total lung was defined as gross tumor minus both lungs; automatic contour extraction was performed using CT‐based thresholds. The RP grade was assigned using the Common Toxicity Criteria for Adverse Events (CTCAE) version 4.0. As DVH parameters, V5, V20, MLD, and VS5 were calculated. The relationships between the incidences of grades ≥ 2 RP and parameters of DVH were analyzed using the log‐rank test for univariate analysis and the Cox proportional hazard model for multivariate analysis. Cutoff values for continuous variables were decided from individual receiver operating characteristic (ROC) curves before performing the log‐rank test. The cutoff values at the maximal point of the Youden Index were adopted. The associations between total lung volume and DVH parameters were tested using the Pearson product‐moment correlation coefficient. Severe RP that would cause permanent treatment discontinuation according to the PACIFIC trial criteria was defined as either grade ≥ 3 or grade 2 that required the maintenance of >10 mg prednisolone for more than 12 weeks.^14^ Because the V20 of the DVH parameters in the PACIFIC trial was below 35%, the rate of severe RP was calculated for patients in our study with V20 values below 35%. The cumulative incidence rate of RP was calculated using the Kaplan‐Meier method; the RP rate was also stratified according to significant factors on univariate analysis. Two‐tailed *P*‐values of <.05 were considered to be significant predictors. All statistical tests were performed using The R software, version 3.2.0 (R Foundation for Statistical Computing).

## 3 RESULTS

Table 1 shows the characteristics of the 45 patients who met the eligibility criteria. The total dose of radiation therapy was 60 Gy in 44 patients and 54 Gy in 1 patient. The median V5, V20, MLD, VS5, and total lung volume were 41.45% (range: 17.86%‐72.59%), 27.48% (range: 10.1%‐48.59%), 14.89 Gy (range: 6.07‐22.62 Gy), 1980.24 cm^3^ (range: 612.30‐3376.51 cm^3^), and 3176.94 cm^3^ (range: 1987.64‐4832.98 cm^3^), respectively. Grades 0, 1, 2, 3, 4, and 5 RP developed in 1 (2.2%), 23 (51.1%), 16 (35.6%), 3 (6.7%), 0 (0.0%), and 2 (4.4%) of the patients, respectively. The median follow‐up period was 20.0 months (range 2.4‐151.8 months).

**Table 1:** Patient characteristics

			%
Age (years)	Median (range)	63 (36‐84)	–
Sex	Male	41	91
	Female	4	9
ECOG‐PS	0	18	40
	1	27	60
Smoking history	Never	1	2
	Former	19	42
	Current	25	56
Lobe^a^	Upper	36	80
	Lower	8	18
Laterality^a^	Right	24	53
	Left	20	44
Histology	Adenocarcinoma	14	31
	Squamous cell carcinoma	27	60
	Non‐small cell carcinoma	4	9
C‐stage	IIIA	7	16
	IIIB	38	84
FEV1 (l)^a^	Median (range)	2.35 (0.48‐4.11)	–
FVC (l)^a^	Median (range)	3.32 (1.51‐6.35)	
%VC (%)	Median (range)	96.9 (62.7‐159.8)	
Total lung volume (cm^3^)	Median (range)	3176.94 (1979‐4833)	–
Tumor size (mm)	Median (range)	55.0 (10.0‐111.0)	–
GTV volume (cm^3^)	Median (range)	167.61 (128.38‐745.31)	–
V5 (%)	Median (range)	41.45 (17.86‐72.59)	–
V20 (%)	Median (range)	27.48 (10.10‐48.59)	–
MLD (Gy)	Median (range)	14.89 (6.07‐22.62)	–
VS5 (cm^3^)	Median (range)	1980.2 (612.3‐3376.5)	–

Abbreviations: %VC, % vital capacity; ECOG‐PS, Eastern Cooperative Oncology Group performance status; FEV1, forced expiratory volume in 1 s; FVC, forced vital capacity; GTV, gross tumor volume; MLD, mean lung dose of the lung; V20, percentage of the lung volume received at least 20 Gy; V5, percentage of the lung volume received at least 5 Gy; VS5, volume of the lung spared from 5 Gy.

^a^ These factors have missing values.

**Figure 1:**
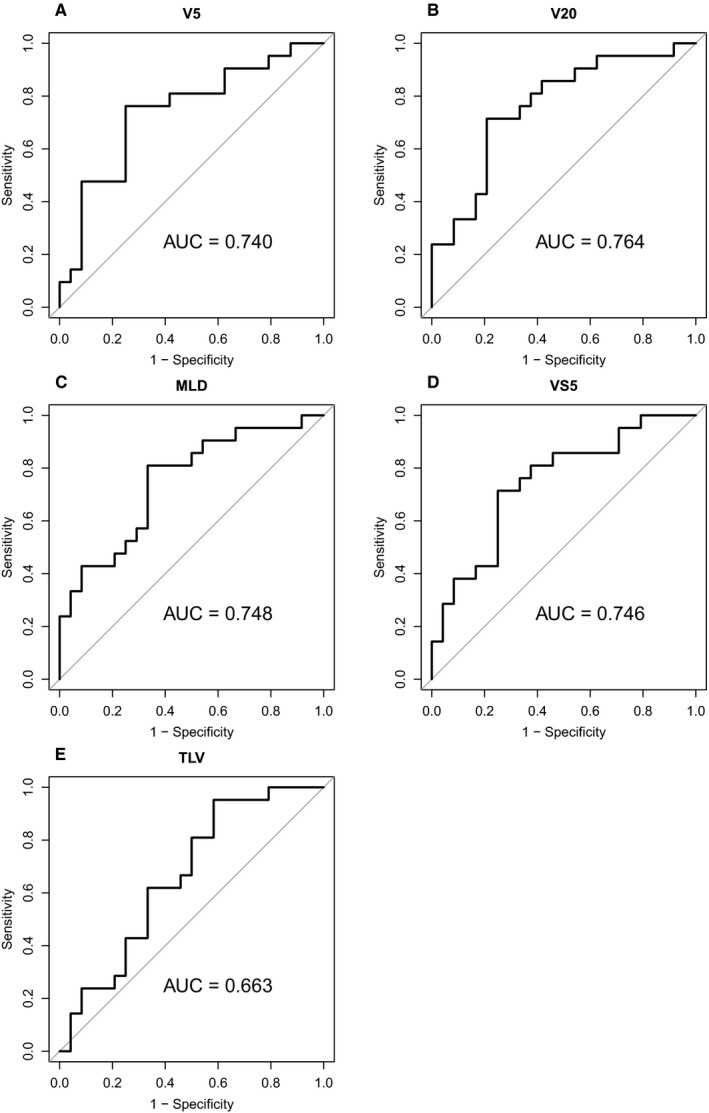
Receiver operating characteristic curves; (A) the percentage of the lung volume that received more than 5 Gy (V5), (B) the percentage of the lung volume that received more than 20 Gy (V20), (C) the mean lung dose (MLD), (D) the lung volume spared from a 5 Gy dose (VS5), and (E) the total lung volume

Figure 1 shows individual ROC curves. In ROC analysis, we found that V5, V20, MLD, VS5, and TLV cutoff values of 41%, 28%, 15 Gy, 1950 cm^3^, and 3680 cm^3^ provided the best combination of sensitivity and specificity. Sensitivity/specificity were 0.762/0.75, 0.714/0.792, 0.81/0.667, 0.714/0.75, and 0.952/0.417, respectively. Results of univariate and multivariate analyses of factors affecting ≥2 RP are shown in Table 2. On univariate analysis, the V5, V20, MLD, VS5, and total lung volume were significant predictors of grade ≥ 2 RP (*P* = .001, .003, .03, .004, and .02, respectively). The absolute Pearson correlation coefficients between V5/V20, V5/MLD, V5/VS5, V20/MLD, V20/VS5, MLD/VS5, and VS5/total lung volume were 0.940, 0.885, 0.732, 0.955, 0.711, 0.706, and 0.753, respectively. In contrast, the correlation coefficients between V5/total lung volume, V20/total lung volume, and MLD/total lung volume were 0.125, 0.155, and 0.186, respectively. From among the V5, V20, MLD, and VS5, we selected V20 for multivariate analysis based on Tsujino et al’s study.^12^ On multivariate analysis, V20 was a significant predictor (*P* = .007), whereas total lung volume was not (*P* = .055) on multivariate analysis..

**Table 2:** Univariate analyses of predictive factors related to grade ≥ 2 radiation pneumonitis

Factor	N	Univariate *P*‐value	Multivariate *P*‐value
Age (years)			
<63	8/20	.3	NE
≥63	13/25		
Sex			
Male	21/41	.09	
Female	0/4		NE
ECOG‐PS			
0	8/18	.6	NE
1	13/27		
Smoking history			
Never/Former	10/20	.6	NE
Current	11/25		
Lobe^a^			
Lower lobe	4/8	.7	NE
Upper lobe	17/36		
Laterality^a^			
Right	13/24	.5	NE
Left	8/20		
FEV1 (l)^a^			
<1.7	1/7	.09	NE
≥1.7	17/31		
FVC (l)^a^			
<3.0	9/15	.3	NE
≥3.0	9/23		
%VC (%)^a^			
<99	10/24	.5	NE
≥99	8/14		
Total lung volume (cm^3^)			
<3680	20/35	.02	.055
≥3680	1/10		
Tumor size (mm)^a^			
<67	18/32	.1	NE
≥67	3/11		
GTV volume (cm^3^)			
<62	13/30	.1	NE
≥62	8/15		
V5 (%)			
<41	5/22	.001	NE
≥41	16/23		
V20 (%)			
<28	6/23	.003	.007
≥28	15/22		
MLD (Gy)			
<15	7/23	.03	NE
≥15	14/22		
VS5 (cm^3^)			
<1950	15/21	.004	NE
≥1950	6/24		

Abbreviations: %VC, % vital capacity; CI, confidence interval; ECOG‐PS, Eastern Cooperative Oncology Group Performance Status; FEV1, forced expiratory volume in 1 s; FVC, forced vital capacity; GTV, gross tumor volume; MLD, mean lung dose of the lung; NE, not entered; V20, percentage of the lung volume received at least 20 Gy; V5, percentage of the lung volume received at least 5 Gy; VS5, volume of the lung spared from 5 Gy.

^a^ These variables have missing values.

**Figure 2:**
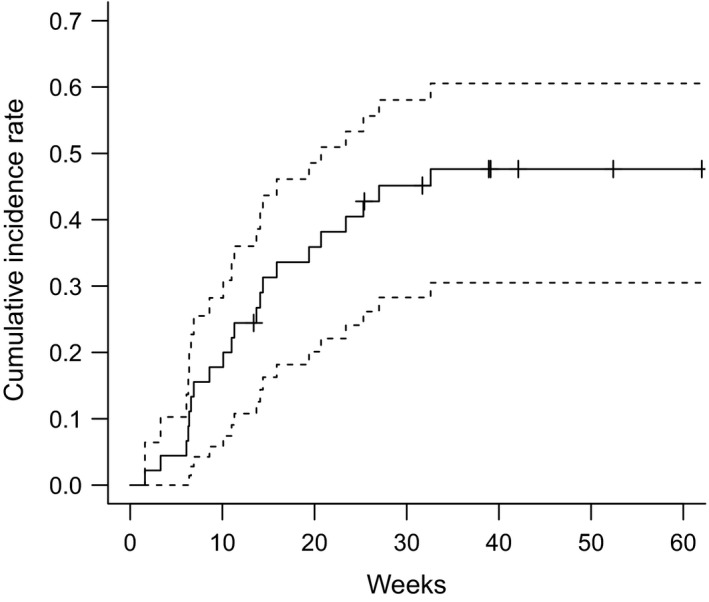
Cumulative incidence rate of grade ≥ 2 radiation pneumonitis after completion of radiotherapy. The dashed lines represent the 95% confidence interval

**Figure 3:**
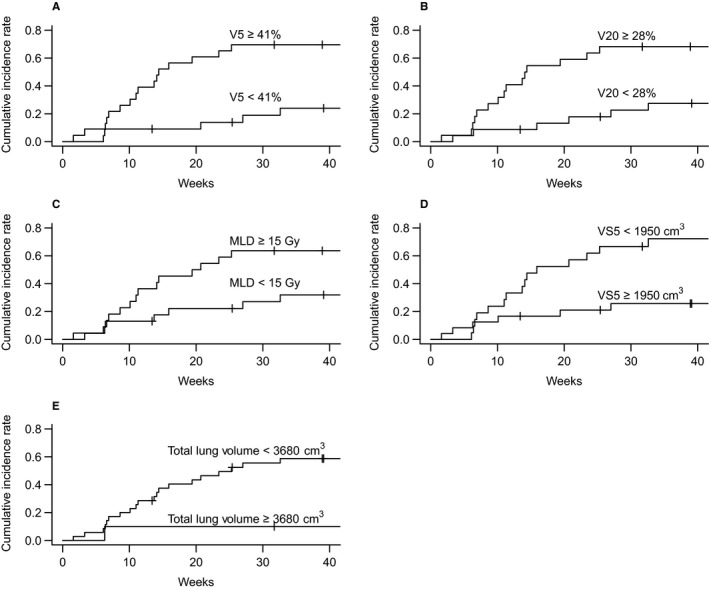
Subgroup analysis of the cumulative incidence rates of grade ≥ 2 radiation pneumonitis after completion of radiotherapy; (A) the percentage of the lung volume that received more than 5 Gy (V5), (B) the percentage of the lung volume that received more than 20 Gy (V20), (C) the mean lung dose (MLD), (D) the lung volume spared from a 5 Gy dose (VS5), and (E) the total lung volume

The median duration between the end of definitive CCRT and the development of grade ≥ 2 RP was 11.29 weeks (range: 1.57‐32.57 weeks). The Kaplan‐Meier curve for the cumulative incidence of grade ≥ 2 RP is shown in Figure 2; incidence rates were 42.8% (95% confidence interval [CI]: 26.2‐55.6%) at 6 months and 47.6% (95% CI: 30.5%‐60.5%) at 12 months. The Kaplan‐Meier curves for the cumulative incidence of grade ≥ 2 RP were stratified according to the five factors that were significant predictors on univariate analysis are shown in Figure 3. The incidence rates of grade ≥ 2 RP after 6 months were as follows: 18.9% and 69.6% in patients with V5 values of < 41% and ≥ 41%, respectively; 17.8% and 68.2% in patients with V20 values of <28% and ≥28%, respectively, 22.2% and 63.6% in patients with MLDs of <15 Gy and ≥15 Gy, respectively; 66.7% and 21.1% in patients with VS5 values of <1950 and ≥1950 cm^3^, respectively, and 66.7% and 21.1% in patients with total lung volumes of <3680 and ≥3680 cm^3^, respectively.

Thirty‐seven of the 45 patients had a V20 of 35% or less. Grades 0, 1, 2, 3, 4, and 5 RP developed in 1 (1.4%), 21 (56.8%), 12 (32.4%), 2 (5.4%), 0 (0.0%), and one (1.4%) of the patients, respectively. Severe RP that would require permanent treatment discontinuation according to the PACIFIC trial criteria developed in 6 (16.2%) of the 37 patients. The V20 cutoff value calculated using the ROC curves was 28.5%; on univariate analysis, V20 was the only significant predictor of severe RP in this subgroup (*P* = .01).

## 4 DISCUSSION

Radiation pneumonitis is a noteworthy adverse event in patients who have undergone definitive radiotherapy for NSCLC; V20 and MLD are major predictors among the various DVH parameters. Graham et al found that V20 was a predictive factor of grades ≥ 2 RP on univariate and multivariate analyses^5^; the incidences of grade ≥ 2 RP at 24 months were 0%, 8%, 13%, and 36% in patients with V20 values of <22%, 22%‐31%, 32%‐40%, and >40%, respectively. Forty‐two percent of patients received neoadjuvant or concurrent chemotherapy. As the incidence of RP appeared to be elevated with concurrent chemotherapy, V20 was related to grade ≥ 2 RP after treatment for definitive CCRT, on univariate analysis in the study by Tsujino et al^6^; the 6‐month cumulative incidences rates of grade ≥ 2 RP were 14% in those with V20 values <26% and 63% in those with V20 values ≥26%. Of the 71 patients undergoing CCRT in their study, 36 (50.7%) received a taxane regimen, 33 of which received carboplatin/paclitaxel. A review of over 70 studies of RP by Marks et al revealed that the V20 and MLD were factors related to this adverse effect; the V20 threshold for limiting the risk of symptomatic RP ≤ 20% was 30‐35%.^9^ The major difference between the third and fourth CTCAE versions is mainly the use of steroids for grading pneumonitis. Dang et al investigated RP in 369 patients with lung cancer using the CTCAE version 4 and found that V20 was a significant predictor associated with RP on univariate analysis.^8^ Consistent with such previous studies, the V20 was found to be a significant predictive factor of RP on univariate and multivariate analyses in our study (*P* = .003 and .007, respectively); the cumulative incidence rates of grade ≥ 2 RP after 6 months were 17.8% and 68.2% in patients with V20 values <28% and ≥28%, respectively. Although the diagnostic criteria and cut‐off values for RP in our study were different, the rates of grade ≥ 2 RP as stratified by V20 was close to that of Tsujino et al^6^

Mean lung dose is a predictive factor of RP in patients who received definitive CCRT. In Barriger et al's study, the incidence rates of grade ≥ 2 RP were 2.2% and 19% in patients whose MLDs are <18 and >18 Gy, respectively.^7^ The MLD threshold for maintaining the risk of symptomatic RP ≤20% was 20‐23 Gy.^9^ In our study, MLD was a significant predictive factor of grade ≥ 2 RP on univariate analysis (*P* = .03). Palma et al also presented MLD to be a predictive factor of RP in patients ≤65 years of age who received CCRT using carboplatin/paclitaxel chemotherapy, according to recursive partitioning analysis; their threshold was 10 Gy.^11^ In the study by Dang et al, MLD was found to be a predictor for RP on univariate and multivariate analyses^8^; MLD of patients with grade 1 RP was 14.15 ± 3.26 Gy, that for patients with grade 2 RP was 17.71 ± 3.28 Gy, and that for patients with grades 3‐5 RP was 21.4 ± 3.48 Gy. MLDs for all patients who developed RP of grades ≥ 2 were, therefore, between 17 Gy and 21 Gy. In our study, the MLD threshold for developing RP, as obtained from the ROC curve, was 15 Gy. Although an accurate comparison was not possible because MLD associated with grade ≥ 2 RP was unknown, and patients other than those who received concurrent chemotherapy were also included in their study, our value was close to theirs.

In a previous analysis of 22 patients who received intensity‐modulated radiotherapy (IMRT), V5 was found to be a prognostic factor for grade ≥ 3 RP.^10^ The incidence of grade ≥ 3 RP RP after 12 months was 2% in patients with V5 values ≤70% but was 21% in those with values >70% (*P* = .017). Similarly, V5 was a significant predictor of RP on univariate analysis in our study. In Yom et al's study, the median V5 was significantly higher in the IMRT group (63%, range: 5%‐98%) than in the 3D‐CRT group (57%, range: 12%‐98%). Moreover the median V20 in the IMRT group (35%, range: 3%‐48%) was lower than that in the 3D‐CRT group (38%, range: 8%‐78%); the MLDs were not significantly different. The incidence rates of grade ≥ 3 RP after 12 months were 8% for patients who received IMRT and 32% for those receiving 3D‐CRT. It was posited that the decrease in V20 contributed to a decrease in the incidence of RP. In a randomized trial by Liao et al comparing the outcomes of passive scattering proton therapy (PSPT) to those of IMRT, PSPT resulted in a lower lung V5‐10 but higher lung V20‐80 than did IMRT. The rates of grade ≥ 3 RP did not differ between the 2 groups.^16^ Like in Tsujino et al’s study, V5 correlated with other DVH parameters in our study; hence, it is necessary to plan treatments while considering all the parameters. Additional research is required to ascertain the effects of low‐dose radiation on the lung and determine the optimal balance between the various DVH parameters that minimizes the risk of PR caused by CCRT with cisplatin/docetaxel.

Tsujino et al investigated the relationship between RP and VS5 and found that a VS5 < 1500 cm^3^ was a significant predictive factor of grade ≥ 3 RP.^12^ The incidences of grade ≥ 3 PR were 7.3% and 50% for patients in whom the VS5 values were ≥1500 and <1500 cm^3^, respectively. Chen et al also showed VS5 to be a predictive factor of grade ≥ 2 RP in patients treated with IMRT.^13^ The incidence rate of grade ≥ 2 RP was significantly lower when VS5 of the ipsilateral lung was ≥564.9 cm^3^ than it was with VS5 values <564.9 cm^3^. VS5 of the ipsilateral lung was a predictor of grade ≥ 2 RP. Chen et al’s study demonstrated the importance of the VS5, even though they included small cell lung cancer patients; moreover, they used the toxicity criteria of the RTOG. In our study, VS5 was also found to be a predictor of grade ≥ 2 PR, considering that VS5 and other parameters may contribute to improving the clinical courses of patients with NSCLC who undergo CCRT with cisplatin/docetaxel.

A multicenter study by Palma et al reported that undergoing chemotherapy was a predictive factor for symptomatic RP; its odds ratio with carboplatin/paclitaxel relative to cisplatin/etoposide was 3.33 (*P* < .001).^11^ Onishi et al showed that the incidence of grade ≥ 3 RP was as high as 47% in patients who received weekly docetaxel and 2‐dimensional radiotherapy.^17^ In Dang et al's study, the incidence rates of grade ≥ 2 were 39.7%, 31%, and 33.6% in patients who received concomitant cisplatin/docetaxel, concomitant cisplatin/vinorelbine, and sequential chemotherapy after radiotherapy; the corresponding incidence rates of grade ≥ 3 RP were 18.4%, 9.5%, and 11.2%, respectively.^8^ The incidence of grade ≥ 3 RP was higher in the concomitant cisplatin/docetaxel group than in the concomitant cisplatin/vinorelbine group. While a comparison of RP incidences between Dang et al’s study and our study should be performed with caution due to the lack of reporting of DVH parameters in their cisplatin/docetaxel group, the incidence rate of grade ≥ 2 RP in our study was 47.6% at 12 months, which was slightly higher than that in their study. IMRT was associated with a lower incidence rate of grade ≥ 3 RP^18^; 22% of the patients in their study received IMRT, which may explain this result. To our knowledge, ours is the first study to show the incidence of grade ≥2 RP and its associated DVH parameters in patients undergoing definitive CCRT with cisplatin/docetaxel.

Programmed death 1 (PD‐l) and PD‐1 ligand 1 (PD‐L1) are important molecules in immune regulation. In comparison with docetaxel, Nivolumab, a PD‐1 inhibitor, improved OS rates in patients previously treated with squamous‐cell lung cancer. Pneumonitis is a serious adverse event caused by the use of PD‐1 inhibitors, leading to the discontinuation of cancer therapy, with resultant mortality.^19^ Durvalumab blocks the interaction between PD‐L1 and PD‐1, but does not bind to PD‐1 ligand 2 (PD‐L2).^20^ Khunger et al showed that the incidence of pneumonitis were 3.6% and 1.3% in the PD‐1 and PD‐L1 inhibitor groups, respectively. The incidence of pneumonitis in patients who received PD‐1 inhibitors was significantly higher than that of PD‐L1 inhibitors.^21^ One reason for the lower rate of pneumonitis in PD‐L1 inhibitors is the presence of PD‐L2. PD‐L2 might play an important role in accommodating immune tolerance in the lungs; it interacts with PD‐l and is affected by PD‐l inhibitors. In a study involving murine models, Xiao et al suggested that PD‐1 blockade may shift the balance of PD‐L2 interaction with binding partners, and increase the capacity of PD‐L2 binding to repulsive guidance molecule b, which is able to lead to pneumonitis.^22^ Further experimental investigations about pneumonitis induced by combination therapy of PD‐L1 inhibitor and radiotherapy are warranted.

Durvalumab cannot be administered if grade ≥ 2 RP occurs.^14^ As such, the management of grade ≥ 2 RP became more urgent after the publication of the PACIFIC trial that showed the benefits of durvalumab treatment after chemoradiotherapy. In the study by Hosoya et al, 19 of 82 patients (23%) became ineligible for durvalumab treatment (as specified by the PACIFIC trial criteria) after receiving CCRT^23^; of these 19 patients, 6 developed grade ≥ 2 RP within 42 days after CCRT. Their study further revealed that old age, male sex, and radiotherapy with a V20 ≥ 35% were related to durvalumab ineligibility after CCRT. In the PACIFIC trial, the hazard ratio for death was 0.42 for patients who commenced treatment with durvalumab < 14 days after completing CCRT, and was 0.81 for those who commenced treatment ≥ 14 days after CCRT.^15^ These data showed that the early introduction of durvalumab was beneficial and that physicians do not have to wait 42 days after CCRT before administering this agent. Pneumonitis had the highest incidence rate of all adverse events that led to the criteria for the discontinuation (4.8% of the patients in the durvalumab group and 2.6% of those in the placebo group) and RP (1.3% and 1.3%, respectively). Additionally, a criterion for the discontinuation of durvalumab in the PACIFIC trial was the occurrence of severe grade ≥ 2 RP that required > 10 mg prednisolone for more than 12 weeks^14^; moreover, the V20 was required to be 35% or lower to avoid the occurrence of RP. In our study, severe RP that met the PACIFIC trial's abovementioned criterion for permanent discontinuation of durvalumab occurred in 6 of 37 patients (16.2%) who had V20 values of ≤35%. Only the V20 was a significant predictive factor of RP on univariate analysis despite the small sample size. In the PACIFIC trial, RP of all grades occurred in 20.2% of patients who received the durvalumab and 15.8% of those who received placebo^15^; hence, durvalumab appeared to increase the risk of pneumonitis slightly. According to our data, the incidence of severe RP is likely to be 16.2% or higher if durvalumab is started immediately after the end of CCRT using cisplatin/docetaxel. Because details regarding radiotherapy in the PACIFIC trial have not yet been published, additional research is required to determine the effects of durvalumab on the lung after CCRT, as well as the associated DVH parameters, in patients who discontinue treatment.

Grade 5 RP developed in two patients in our study (4.4%). In previous prospective trials, the V20 value was not among the eligibility criteria. Grade 5 RP occurred in 1.9% of the patients undergoing CCRT with carboplatin/paclitaxel in the West Japan Thoracic Oncology Group 0105 trial^2^ and 2.0% of those undergoing the same treatment in the OLCSG 0007 trial.^1^ In recent prospective trials, the V20 has been limited to 35% or less. Two of the 283 patients (0.7%) in the CCRT with cisplatin/pemetrexed group of the PROCLAIM study died of possible RP, while no grade 5 RP occurred among patients receiving CCRT plus cisplatin/etoposide.^3^ Although the incidence of grade 5 RP appeared to have decreased slightly after restricting the V20 values, grade 5 RP still occurred in 5.3% and 1.9% of patients administered cisplatin/S‐1 and cisplatin/vinorelbine in the West Japan Oncology Group 5008L study of Japanese patients.^4^ Hence, Japanese patients receiving CCRT should be monitored for grade 5 RP, as risk factors other than the DVH parameters may exist (this requires additional investigation).

To the best of our knowledge, ours is the first study to determine the predictive factors for grade ≥ 2 or severe RP that would require permanent treatment discontinuation under the PACIFIC trial criteria in patients administered CCRT with cisplatin/docetaxel. There were several limitations in our study. First, this was a retrospective analysis with small sample size. Therefore, more studies with more lung cancer patients receiving CCRT with cisplatin/docetaxel are needed to confirm the prognostic value of V20. Second, assessments of important factors, such as single nucleotide polymorphisms and background lung disease, were not performed. For example, Xu et al^24^ reported that genetic variants of surfactant protein D were associated with the development of RP. Third, we only evaluated RP but not other types of adverse events that could also lead to permanent treatment discontinuation according to the PACIFIC trial criteria.

## 5 CONCLUSION

V20 is a significant predictor of grade ≥ 2 and severe RP. Severe RP that would lead to permanent discontinuation of treatment according to the PACIFIC trial criteria developed in 16.2% of the patients who received definitive CCRT with cisplatin/docetaxel. Detailed future investigations of the relationship RP and parameters of DVH in patients who receive durvalumab as part of a concurrent chemotherapy regimen are warranted.

## CONFLICT OF INTERESTS

KK1 received honoraria from AstraZeneca, outside the submitted work. KK1 belongs to the donation course funded by Tsuyama Chuo Hospital; KK2 received honoraria from AstraZeneca outside the submitted work.

## AUTHORS' CONTRIBUTIONS

KK1 participated in the design of the study, performed treatment, collected the data, and drafted the manuscript; TO participated in the design of the study, performed treatment, and performed statistical analysis; KW participated in the design of the study, collected the data, and performed treatment; NK, ST, and KK2 participated in the design of the study and performed treatment; MK, TH, YM, and SK participated in the design of the study. All authors read and approved the final manuscript.

## ETHICS APPROVAL AND CONSENT TO PARTICIPATE

The institutions’s review board approved this study (approval number: 1809‐018). Written informed consent was obtained prior to treatment. The choice to opt‐out was provided through notifications displayed on the hospital’s website and outpatient ward before the start of this study.

## Data Availability

The institution's review board prohibits data sharing.
